# Supplementary material for: Controlled filamentation instability as a scalable fabrication approach to flexible metamaterials
Source: Nat Commun. 2022 Oct 18;13:6154. doi: 10.1038/s41467-022-33853-1 (PMC9579152; doi:10.1038/s41467-022-33853-1)
Supplement: Supplementary file 1 — Supplementary Information [file 41467_2022_33853_MOESM1_ESM.pdf]

SUPPLEMENTARY INFORMATION

Controlled filamentation instability as a scalable fabrication approach to flexible metamaterials

Authors : William Esposito[1], Louis Martin-Monier[1], Pierre-Luc Piveteau[1], Bingrui Xu[2], Daosheng Deng[3]*, Fabien Sorin[1]*

[1]Laboratory for Photonic and Fiber devices, Ecole Polytechnique Fédérale de Lausanne (EPFL), Lausanne, Switzerland

[2]Department of Basic Courses, Naval University of Engineering, Wuhan, China

[3]Department of Aeronautics and Astronautics, Fudan University, Shanghai, China

*Correspondence to: dsdeng@fudan.edu.cn and fabien.sorin@epfl.ch

Keywords: Nanowire array, Templated instabilities, Chalcogenide Glass, Thermal Drawing, Filamentation, Diffraction grating, Metasurface

Table of Contents

Note 1. PVD on square substrates 3

Note 2. Glancing off-axis angle PVD geometry 3

Note 3. ChG film encapsulation and preform consolidation 4

Note 4. Increasing the varicose mode initial amplitude by open-air reflow 4

Note 5. Experimental in-situ observation of the dewetting process 5

Note 6. Linearized model details 6

Anisotropic perturbation growth 6

Initial amplitude ratio estimation 8

Simulation parameters 8

Note 7. Body force from a reconstructed Van der Waals potential for a corrugated free film 8

Development of the flat film contribution 10

Development of the corrugation contribution 12

Total contributions 14

CFD model: introduction 15

CFD model: disjoining pressure models 15

Note 8. CFD model sensitivity analysis 16
Mesh and time discretization 16
Draw ratio 17
Interfacial energy 17
Film thickness 18
Note 9. Experimental validation of the critical $R_{height,0}$ for filamentation 18
Note 10. Diameter distribution 20
Note 11. Diffraction setup 21
Note 12. Rigorous coupled-wave analysis (RCWA) 22
Note 13. Scattering response of NWs 22

Note 1. PVD on square substrates

Our model predicts the evolution of a viscous film's thickness fluctuations under anisotropic stretching. We demonstrated the validity of this theory for predicting capillary break-up dynamics for sinusoidal perturbations, but other templated dewetting geometries could be investigated[1]. We envision that sharper substrate features at precise locations could provide an even stronger localized drive to dewetting (higher $P_L = \gamma\kappa$). However, deposition onto a square template without an intermediate substrate reflow step resulted in a peculiar film geometry. The film was 9 times thicker at the top of the squares than on the groove walls, and 6 times thicker at the bottom of the grooves, as can be seen on Supplementary Fig. S1. Such a geometry doesn't lend itself to regular periodicity but could result in having sub-periodicity with one small and one big NW given by the top and bottom horizontal surfaces, as shown in Fig. 3 (main text).

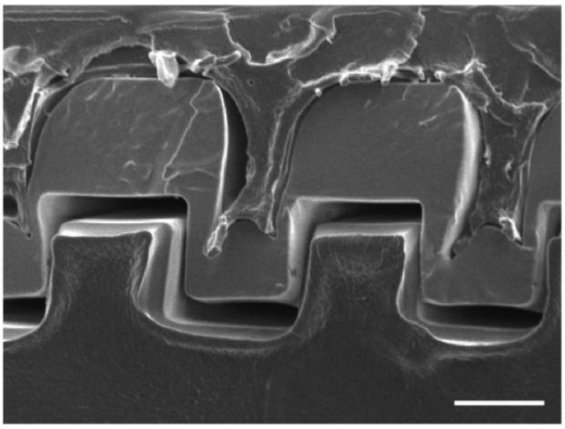

Supplementary Fig. S1 | SEM image of a cross-section obtained by breaking an embedded ChG film in liquid nitrogen. PVD was performed directly on a square template, without an intermediate reflow step of the substrate. Scale bar: 4 µm.

Note 2. Glancing off-axis angle PVD geometry

The deposition of a ChG layer on the patterned polymer films is performed on a rotating platform. This provides uniformity between different samples placed at the same radius. However, the source is place off-rotating axis so that an *effective* deposition angle must be calculated. Additionally, to tilt the sample further to the desired angle, pyramidal scaffolds (3D printed) are inserted between the rotating sample platform and the polymer films. The deposition angle, sum of the scaffold angle and evaporation angle, can therefore be calculated as being between -34° and 83°, or 24° on average. The two extreme positions of a sample placed in such a way are represented in Supplementary Fig. S2. The deposition thickness was calibrated by placing a Silicon fragment on the scaffold, beside the polymer film, and measuring the depth of a scratch with an optical profilometer.

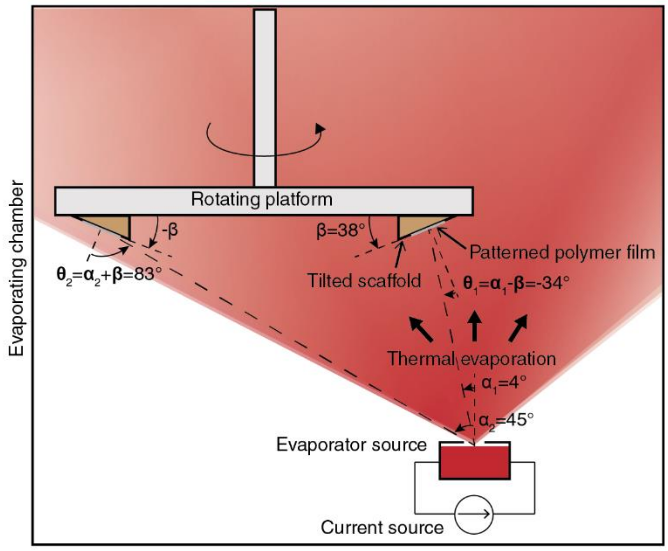

Supplementary Fig. S2 | Thermal evaporation geometry and setup. Average deposition angles were measured experimentally and adjusted using tilted scaffolds.

Note 3. ChG film encapsulation and preform consolidation

In the process described in the main text Fig. 1a, the final and most critical step of preform preparation is encapsulation of the deposited ChG film into another layer of the polymeric substrate material, done either by (i) consolidation (annealing under light pressure to ensure intimate conformity between film layer and cladding), or (ii) by solvent casting of the encapsulating material. A key feature of our process is its ability to suppress the instabilities along the fiber axis and ensure a homogeneous filamentation solely during *anisotropic stretching*[2] (c.f. main text Fig. 1b). In both cases, it is therefore important to limit dewetting before the assembly is downscaled, when the film might be prone to dewetting both along and perpendicularly to the fiber axis[1]. Limiting nucleation and growth of holes prior to stretching incentivizes the use of films with minimal initial thickness beyond a critical threshold (~300 nm), which can also pose a limit to the maximal evaporating angle one can resort to. While consolidation implies a longer nucleation and growth of hole stage, dip-coating of thick encapsulating layers often leaves significant residual stress within the encapsulating material, which may lead to delamination. In the rest of this paper, we resort solely to the use of the consolidation technique, which is the most practical. Furthermore, we show in Supplementary Note 4Note 4 that when annealing the sample for consolidation, before the top layer softens and encapsulates the ChG film, open-air reflow can be beneficial to increasing templated film thickness fluctuations.

Note 4. Increasing the varicose mode initial amplitude by open-air reflow

As detailed in the main text, capillary break-up of the deposited film occurs because of the disjoining pressure resulting from thickness variations (varicose mode). To induce a templated amplitude large enough to overcome the spontaneous “Rayleigh-type” instability, one needs to impart an initial thickness fluctuation at the desired wavelength. When depositing the ChG orthogonally to the templated substrate, the film thickness is even. Such a sinuous mode for a glass film in a polymer matrix only reflows and flattens out under Laplace pressure. An

alternative to depositing at an angle which was explored experimentally could be to induce some open-air reflow by isothermal annealing of the ChG film right after deposition, as opposed to inside the fiber cladding during drawing. The two interfaces would then not be symmetrical. The top one would be between air and the ChG (air-fluid), the bottom one between the ChG and the polymer substrate (fluid-fluid). We expect the bottom interface to have a stronger interfacial tension, therefore reflowing and flattening faster than the top one. To verify this assumption, different annealing times were used for similar samples. Cross-sections were then prepared for optical characterization of the film geometry. Supplementary Fig. S3 shows a film after deposition, before and after open-air annealing (15 mns at 220°C) on a hot plate. Although this annealing step could have been used in our process, we preferred to rely solely on PVD at an angle, for three reasons. First, isothermal annealing introduces an additional step. Second, thermal processes are challenging to reproduce consistently. External temperature, air convection, or polymer film contact with the heating device may vary slightly from sample to sample, a few degrees difference resulting in a large viscosity discrepancy, challenging to assess. Finally, and most importantly, isothermal annealing may lead to the appearance of holes in the film wherever imperfection, dust, or any irregularities were present. In the absence of stretching (i.e. drawing), instabilities along the lines (axial) are not dampened enough compared to those across the lines (transverse) to ensure that capillary break-up only happens in the desired direction.

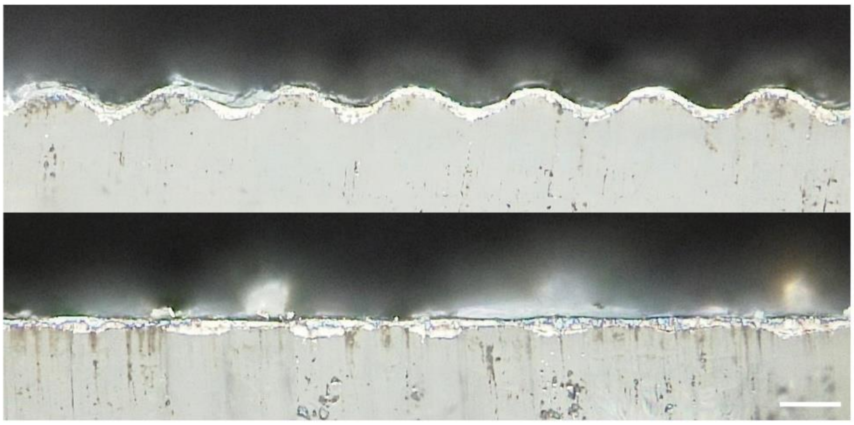

Supplementary Fig. S3 | Extended depth-of-field cross-section micrographs of As_2Se_3 films on a PEI substrate. The top (resp. bottom) image is taken before (resp. after) open-air reflow at 220°C for 15 mns. The top interface flattens faster than the bottom one, leading to a strong thickness fluctuation. Scale bar: 20 µm.

Note 5. Experimental in-situ observation of the dewetting process

By interrupting fiber drawing abruptly, surrounding air cools down the ChG film and its cladding fast (~ 20°C/s), effectively quenching it for observation in situ. Starting from the continuous film, as deposited after PVD and preform consolidation, we were able to identify three stages in the three-dimensional capillary break-up process, complementing the cross-section description and images shown in the main text. A more detailed overview of the break-up process based on optical micrography is shown in Supplementary Fig. S4. First, holes nucleate in the film wherever nanoscopic imperfections or roughness lead to a slightly higher thickness fluctuation. Secondly, assisted by anisotropic stretching, these holes grow along the lines, following areas of smaller thickness. Third, in between holes, the film reflows laterally into long, regularly spaced cylindrical filaments. As apparent in the topmost micrographs, filamentation does not occur regularly over the entire film. Conversely, very regular filaments can be observed once the fiber is scaled down. This observation can be directly linked to the

presence/absence of anisotropy in the dewetting process. Prior to stretching, the film is mobile, and dewetting is initiated without anisotropy along the fiber axis. During downscaling (bottom micrographs in Supplementary Fig. S4), growth of longitudinal instabilities is inhibited while that of transversal instabilities is enhanced, which leads to the filamentation dewetting pattern observed. The steady-state and capillary break-up pathway remain the same for any cross-section considered, which confers stability and reproducibility to our process. The top view, however, does not capture precise thickness fluctuation, as it only discriminates between the continuous ChG film and holes. For a dynamic visualization of thickness fluctuations, one needs to look at a cross-section as it travels through the furnace, as captured by our CFD model.

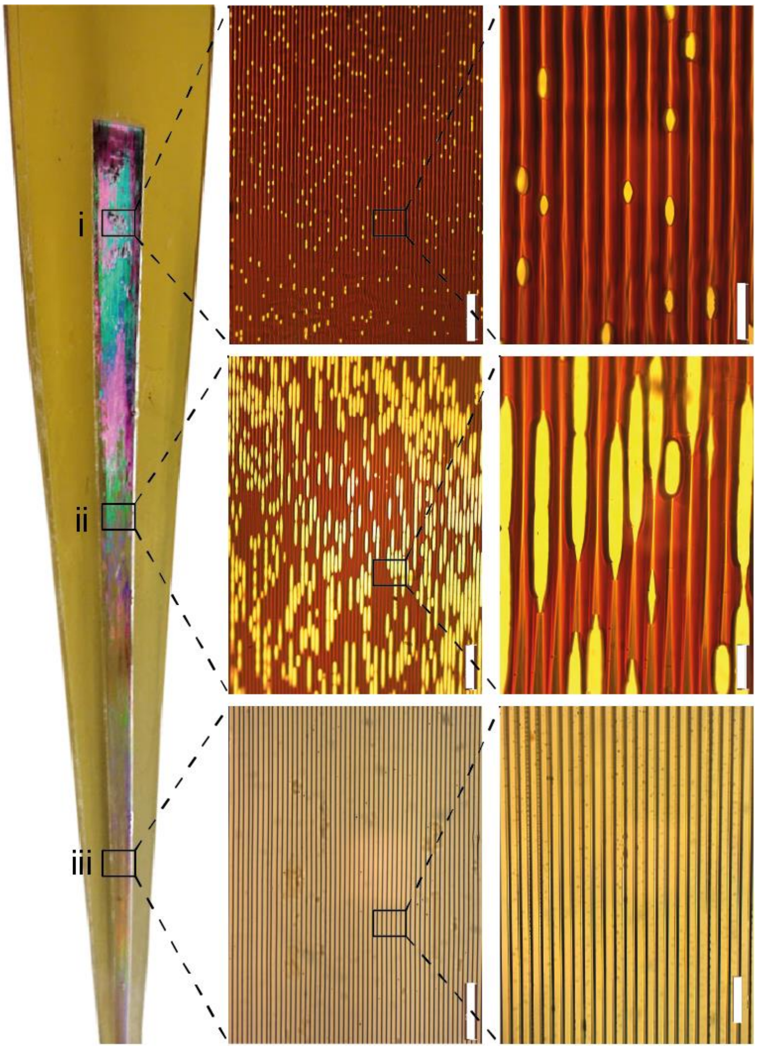

Supplementary Fig. S4 | In situ top-view transmission micrographs of the capillary break-up process unfolding during thermal drawing in the neck-down region. Scale bars: first zoom-in level (left column): 200 µm, second zoom-in level (right column): 50 µm.

Note 6. Linearized model details

Anisotropic perturbation growth

Let us consider a varicose perturbation, with a wavenumber k, a growth rate ω_k, and an initial amplitude perturbation a_0, its amplitude a growing with time t as a continuous function: $a(\mathrm{t})=a_0\ exp(\omega_k\ t)$. The

stretching-induced *anisotropy* entails modified wavenumbers and thus growth rates for instabilities parallel and perpendicular to the stretching direction[2]. With this in mind, the wavenumber $k(t)$ of a given perturbation now depends on time, and so does its growth rate $\omega_k(t)$. Let $K = kH$ be an dimensionless wavenumber to account for downscaling of the film thickness H throughout fiber drawing[3,4]. By considering the equivalent one-dimensional "fiber limit" case for volume conservation[5], we get[2]:

$$k_{\parallel}(t) = \frac{k_{\parallel}(t=0)}{DR(t)^2} \tag{1}$$

$$k_{\perp}(t) = k_{\perp}(t=0)\, DR(t) \tag{2}$$

One can then express dimensionless wavenumbers perpendicular ($K_{\perp}$) and parallel ($K_{\parallel}$) to the fiber axis:

$$K_{\parallel}(t) = \frac{k_{\parallel}(t=0)}{DR(t)^2}\frac{H(t=0)}{DR(t)} = \frac{K_{\parallel}(t=0)}{DR(t)^3} \tag{3}$$

$$K_{\perp}(t) = k_{\perp}(t=0)\, DR(t)\, \frac{H(t=0)}{DR(t)} = K_{\perp}(t=0)\ (independent\ of\ DR) \tag{4}$$

These two equations denote a distinct evolution along and perpendicular to stretching. Along the longitudinal direction, as DR(t) increases with drawing time, the wavenumber of a given perturbation is continuously reduced, which disfavors the growth of instabilities. We now show why a reduction in all $K_{\parallel}$ by stretching leads to a decrease in the *amplitude* growth, compared to the transverse case.

Let us consider that all wavenumbers are initially present in infinitesimal amplitudes. At any given time t of drawing there is a wavenumber $K_{\perp,max}(t)$ (resp. $K_{\parallel,max}(t)$) for which the growth rate $\omega_{K_{\perp,max}}(t)$ (resp. $\omega_{K_{\parallel,max}}(t)$) is maximal. In the transverse case, since $K_{\perp}(t) = K_{\perp}(t=0)$, there is a single $K_{\perp,max}$ for all times t. Therefore, $a_{\perp}(t) = a_0 \exp(\omega_{K_{\perp}} t)$ is maximal for this wavenumber $K_{\perp,max}$:

$$a_{\perp,max}(t) = a_0 \exp(\omega_{K_{\perp,max}} t) \tag{5}$$

However, in the longitudinal case, $a_{\parallel}$ for a given perturbation $K_{\parallel}(t) = \frac{K_{\parallel}(t=0)}{DR(t)^3}$ (i.e. given by the initial perturbation $K_{\parallel}(t=0)$) must be calculated over the draw: $a_{\parallel}(t) = a_0 \exp(\Omega_{K_{\parallel}}(t)t)$ where $\Omega_{K_{\parallel}}(t) = \frac{1}{t}\int_0^t \omega_{K_{\parallel}}(\tau)d\tau$ is the total (integrated) growth rate through thermal drawing. As opposed to the transverse case, maximal amplitude must therefore be calculated over all initial instabilities $a_{\parallel,max}(t) = \max_{K_{\parallel}(t=0)}\left(a_0 \exp\left(\Omega_{K_{\parallel(t=0)}}(t)t\right)\right) = \max_{K_{\parallel}(t=0)}[a_0 \exp(\int_0^t \omega_{K_{\parallel(t=0)}}(\tau)\, d\tau)]$, and since at all times τ : $\omega_{K_{\parallel(t=0)}}(\tau) \leq \omega_{K_{\parallel,max}}(\tau)$, longitudinal amplitude growth is suboptimal:

$$a_{\parallel,max}(t) \leq a_0 \exp(\omega_{K_{\parallel,max}} t) \tag{6}$$

Compared to Rayleigh-Plateau's non-stretched case[3,6], drawing therefore dampens the fastest growing instability in the longitudinal direction.

Initial amplitude ratio estimation

As in the model for *anisotropic instability of a stretching viscous sheet (AISVS)*[2], we consider the perturbation corresponding to the maximal total growth rate Ω_{max}[3,4]. We now compare it to the templated total growth rate Ω_T (for $\lambda_\perp = P$) and therefore its amplitude a_T to that of the fastest-growing instability a_{max}. Fig. 2c (main text) compares Ω_T at three template periodicities to Ω_{max} for different initial film thicknesses H_0. Parameters used for this numerical estimation can be found below. If the amplitude of the fastest-growing perturbation at the end of the draw remains smaller than a templated one (despite growing faster), the latter dominates the instability and break-up. This provides the intended periodicity**.** For such a condition to be met, the initial amplitude ratio (at t = 0) $R_{initial} = \frac{a_{T,initial}}{a_{max,initial}}$ should be large enough. The final amplitude at the end of drawing is given by the initial amplitude and the total growth over drawing. A conservative (sufficient, although not necessary) condition is that at the end of the draw (and thus at all times), i.e. t = t_f, the ratio:

$$R_{final} = \frac{a_{T,final}}{a_{max,final}} = \frac{a_{T,initial}\exp\left(\Omega_{T,final}t_f\right)}{a_{max,initial}\exp(\Omega_{max,final}t_f)} = R_{initial}R_{growth,final} \tag{7}$$

should be larger than 1, with $R_{growth,final} = \exp\left(\left[\Omega_{T,final} - \Omega_{max,final}\right] t_f\right)$ the relative amplitude growth of an instability at the end of the draw. For capillary break-up to follow the template, this therefore requires $R_{growth,final} > \frac{1}{R_{initial}}$.

We measure from image analysis on SEM cross sections initial varicose amplitudes ~600 nm for deposited templated films. We consider $a_{max,initial}$ to be at most given by the roughness of a film as deposited. This is a conservative assumption, as the film reflows during preform annealing. An AFM measurement can be found in Supplementary Fig. S20 showing peak-to-trough roughness of at most 6 nm, i.e. $a_{max,initial} \leq 6$ nm. Based on these experimental observations, we consider $R_{initial}$ to be typically 100 before drawing.

Simulation parameters

The linearized model used here[2] simulated templated instability growth with the following simulation parameters:

Top layer pressure (Pa.s)	Bottom layer pressure (Pa.s)	γ Interfacial tension ($N.m^{-1}$)	Hamaker constant (J)	Drawing length L (m)	V_{feed} ($m.s^{-1}$)	Draw Ratio
10^5	10^5	0.03	10^{-17}	0.2	$1.6\ 10^{-5}$	30

Supplementary Table S1 | Parameters used in the linearized model numerical calculation

Note 7. Body force from a reconstructed Van der Waals potential for a corrugated free film

Using the Hamaker procedure to obtain the body force potential has two advantages: (i) in contrast to the disjoining pressure approach it can easily be formulated for any interfacial corrugated state of the film's interfaces and is therefore applicable to both short and long wavelength disturbance regimes; (ii) it is computationally easier to formulate the Hamaker potential for an unsymmetrical film system than it is to calculate the disjoining pressure

of such a system. Details on how to implement the body force for Van Der Waals (VdW) interactions based on the general case of a corrugated interface are provided below. Electromagnetic retardation effects, which may reduce the strength of VdW interactions with increased interatomic distance, are here neglected at this stage for the sake of simplicity. The body force is simply the opposite of the gradient of this potential. This derivation stems from (ref.[7–9]). The result for the VdW potential depends on the region of study. The change in potential energy is derived by first computing the potential energy of interaction (due to the VdW force) of molecules in an infinitesimal volume of continuum with respect to (i) the rest of the molecules in the film system and (ii) the rest of the molecules in the infinite system. The subtraction between these two energies is termed the excess VdW potential; the negative gradient of this potential is the body force which is introduced into the equation of motion.

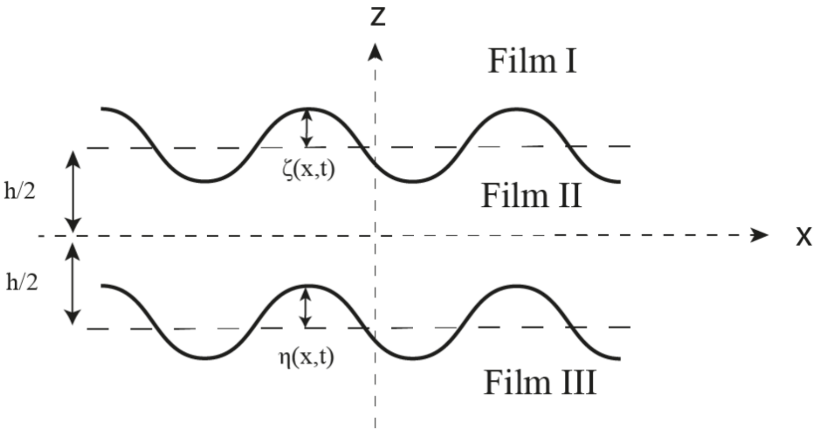

Supplementary Fig. S5 | 2D schematic of the film system. The origin of the system is taken as the midplane of the unperturbed film. Let h be the mean thickness of film II. ζ and η are interface corrugations. Adapted from (ref.[8]).

The VdW potential $w_{i,j}$ between two molecules i and j can be written in the far field approximation as:

$$w_{i,j}(r) \sim \frac{C_{i,j}}{r^6} \tag{8}$$

$$A_{i,j} = \pi^2 \rho^i \rho^j C_{i,j} \tag{9}$$

$F_{i,j}(s)$ represents the potential energy (per unit volume) at a point r in phase i due to an infinite plane of phase j that is located at a perpendicular distance s from r (valid in 3D):

$$F_{i,j}(s) = \rho^i \rho^j \int_s^{\infty} 2\pi r w_{i,j}(r) dr = \frac{\rho^i \rho^j C_{i,j} 2\pi}{4 s^4} = \frac{1}{2\pi} \frac{A_{i,j}}{s^4} \tag{10}$$

Where $A_{i,j} = \pi^2 \rho^i \rho^j C_{i,j}$. The function $H^{\mathbf{1}}{}_{i,j}(x, y, z, t)$ (resp. $H^{\mathbf{2}}{}_{i,j}(x, y, z, t)$) is the perturbation in the potential energy (per unit volume) at a point in phase i, with coordinates (x,y) and located at a distance |z| from the unperturbed upper (resp. lower) interface, caused by the displacement of phase j with the corrugation of the interface.

$$H^{\mathbf{1}}{}_{i,j}(x,z,t) = \rho^i\rho^j \int_{\beta=-\infty}^{\infty}\int_{\alpha=-\infty}^{\infty}\int_{\gamma=0}^{\xi(\alpha,t)} w_{i,j}\big(((z-\gamma)^2+(x-\alpha)^2+(y-\beta)^2)^{1/2}\big)d\beta d\alpha d\gamma \quad (11)$$

$$H^{\mathbf{2}}{}_{i,j}(x,z,t) = \rho^i\rho^j \int_{\alpha=-\infty}^{\infty}\int_{\beta=-\infty}^{\infty}\int_{\gamma=0}^{\eta(\alpha,t)} w_{i,j}\big(((z-\gamma)^2+(x-\alpha)^2+(y-\beta)^2)^{1/2}\big)d\beta d\alpha d\gamma \quad (12)$$

Developing these terms using the potential yields (in 2D):

$$H^{\mathbf{1}}{}_{i,j}(x,z,t) = \rho^i\rho^j \int_{\beta=-\infty}^{\infty}\int_{\alpha=-\infty}^{\infty}\int_{\gamma=0}^{\xi(\alpha,t)} \frac{C_{i,j}}{((z-\gamma)^2+(x-\alpha)^2+(y-\beta)^2)^3}d\alpha d\beta d\gamma \quad (13)$$

$$H^{\mathbf{2}}{}_{i,j}(x,z,t) = \rho^i\rho^j \int_{\beta=-\infty}^{\infty}\int_{\alpha=-\infty}^{\infty}\int_{\gamma=0}^{\eta(\alpha,t)} \frac{C_{i,j}}{((z-\gamma)^2+(x-\alpha)^2+(y-\beta)^2)^3}d\alpha d\beta d\gamma \quad (14)$$

Development of the flat film contribution

For any point in Film I (top layer):

$$\begin{aligned}\rho^I W^I{}_{NP}(x,z,t) = &\int_{z-h/2}^{\frac{h}{2}+z} F_{I,II}(u)\,du + \int_{\frac{h}{2}+z}^{\infty} F_{I,I}(u)\,du + \int_{0}^{\infty} F_{I,I}(u)\,du + \int_{z-\frac{h}{2}}^{0} F_{I,I}(u)\,du \\ &-\left(\int_{z-\frac{h}{2}}^{\infty} F_{I,II}(u)\,du + \int_{0}^{\infty} F_{I,I}(u)\,du + \int_{z-\frac{h}{2}}^{0} F_{I,I}(u)\,du\right) \\ &-\left(\int_{z+\frac{h}{2}}^{\infty} F_{I,II}(u)\,du + \int_{0}^{\infty} F_{I,I}(u)\,du + \int_{z+\frac{h}{2}}^{0} F_{I,I}(u)\,du\right)\end{aligned} \quad (15)$$

where NP stands for not perturbed, i.e. the flat film situation. After simplification:

$$\boldsymbol{\rho^I W^I{}_{NP}(x,z,t) = \frac{1}{2\pi}\left(A_{I,I} + A_{II,II} - 2A_{I,II}\right)\frac{1}{3\left(\frac{h}{2}+z\right)^3}} \quad (16)$$

For any point in Film II :

$$\rho^{\mathrm{II}}W^{II}{}_{NP}(x,z,t)=\left(\int_{\frac{h}{2}-z}^{\infty}F_{II,I}(u)\,du+\int_{-\infty}^{z+\frac{h}{2}}F_{II,I}(u)\,du+\int_{0}^{\frac{h}{2}-z}F_{II,II}(u)\,du+\int_{z+\frac{h}{2}}^{0}F_{II,II}(u)\,du\right)$$ (17)

$$-\left(\int_{-\infty}^{\frac{h}{2}+z}F_{II,I}(u)\,du+\int_{\frac{h}{2}+z}^{0}F_{II,II}(u)\,du+\int_{0}^{\infty}F_{II,II}(u)\,du\right)$$

$$-\left(\int_{0}^{\frac{h}{2}-z}F_{II,II}(u)\,du+\int_{-\infty}^{0}F_{II,II}(u)\,du+\int_{\frac{h}{2}-z}^{\infty}F_{II,I}(u)\,du\right)$$

$$\boldsymbol{\rho^{\mathrm{II}}W^{II}{}_{NP}(x,z,t)=-\int_{-\infty}^{\infty}(F_{II,II}(u))\,du}$$ (18)

For any point in Film III (bottom layer):

$$\rho^{III}W^{III}{}_{NP}(x,z,t)=\int_{0}^{\infty}F_{I,I}(u)\,du+\int_{0}^{-\frac{h}{2}-z}F_{I,I}(u)\,du+\int_{-\frac{h}{2}-z}^{\frac{h}{2}-z}F_{I,II}(u)\,du+\int_{\frac{h}{2}-z}^{\infty}F_{I,I}(u)\,du$$

$$-\left(\int_{0}^{\infty}F_{I,I}(u)\,du+\int_{0}^{-\frac{h}{2}-z}F_{I,I}(u)\,du+\int_{-\frac{h}{2}-z}^{\infty}F_{III,III}(u)\,du\right)$$ (19)

$$-\left(\int_{0}^{\infty}F_{II,II}(u)\,du+\int_{0}^{\frac{h}{2}-z}F_{II,II}(u)\,du+\int_{\frac{h}{2}-z}^{\infty}F_{I,II}(u)\,du\right)$$

$$\boldsymbol{\rho^{III}W^{III}{}_{NP}(x,z,t)=\left(A_{I,I}+A_{II,II}-2A_{I,II}\right)\frac{1}{3\left(\frac{h}{2}-z\right)^{3}}}$$ (20)

To obtain the body force, we can derivate along relevant directions:

- Derivative along x

$$-\frac{\partial\rho^{I}W^{I}{}_{NP}(x,z,t)}{\partial x}=0$$ (21)

$$-\frac{\partial\rho^{II}W^{II}{}_{NP}(x,z,t)}{\partial x}=0$$ (22)

$$-\frac{\partial\rho^{III}W^{III}{}_{NP}(x,z,t)}{\partial x}=0$$ (23)

- Derivative along z

$$-\frac{\partial \rho^I W^I{}_{NP}(x,z,t)}{\partial z} = \frac{1}{2\pi}\left(A_{I,I} + A_{II,II} - 2A_{I,II}\right)\frac{1}{\left(\frac{h}{2}+z\right)^4} \tag{24}$$

$$-\frac{\partial \rho^{II} W^{II}{}_{NP}(x,z,t)}{\partial z} = 0 \tag{25}$$

$$-\frac{\partial \rho^{III} W^{III}{}_{NP}(x,z,t)}{\partial z} = -\frac{1}{2\pi}\left(A_{I,I} + A_{II,II} - 2A_{I,II}\right)\frac{1}{\left(\frac{h}{2}-z\right)^4} \tag{26}$$

Development of the corrugation contribution

Let us calculate the film I excess VdW potential considering that the reference system is the corrugated interface (I, II) with the interface (II, III) at infinity. The function $H^1{}_{i,j}(x,y,v,t)$ (resp. $H^2{}_{i,j}\ (x,y,v,t)$) is the perturbation in the potential energy (per unit volume) at a point in phase i, with coordinates (x, y) and located at a distance $|v|$ from the unperturbed upper (lower) interface, caused by the displacement of phase j with the corrugation of the interface

$$\rho^I W^I{}_P(x,z,t) = H^1{}_{I,II}\left(x,-z+\frac{h}{2},t\right) - H^1{}_{I,I}\left(x,-z+\frac{h}{2},t\right) + H^2{}_{I,I}\left(x,z+\frac{h}{2},t\right) - H^2{}_{I,II}\left(x,z+\frac{h}{2},t\right) - \left(H^1{}_{I,II}\left(x,-z+\frac{h}{2},t\right) - H^1{}_{I,I}\left(x,-z+\frac{h}{2},t\right)\right) - \left(H^2{}_{II,I}\left(x,z+\frac{h}{2},t\right) - H^2{}_{II,II}\left(x,z+\frac{h}{2},t\right)\right) \tag{27}$$

where P stands for the perturbed case with respect to the flat film case, i.e. the additional corrugation. This further simplifies:

$$\boldsymbol{\rho^I W^I{}_P(x,z,t) = H^2{}_{I,I}\left(x,-z+\frac{h}{2},t\right) + H^2{}_{II,II}\left(x,-z+\frac{h}{2},t\right) - 2H^2{}_{I,II}\left(x,-z+\frac{h}{2},t\right)} \tag{28}$$

$$\rho^{II} W^{II}{}_P(x,z,t) = -H^1{}_{I,II}\left(x,-z+\frac{h}{2},t\right) + H^1{}_{II,II}\left(x,-z+\frac{h}{2},t\right) - H^2{}_{II,II}\left(x,z+\frac{h}{2},t\right) + H^2{}_{I,II}\left(x,z+\frac{h}{2},t\right) - \left(-H^1{}_{I,II}\left(x,-z+\frac{h}{2},t\right) + H^1{}_{II,II}\left(x,-z+\frac{h}{2},t\right)\right) - \left(-H^2{}_{II,II}\left(x,z+\frac{h}{2},t\right) + H^2{}_{I,II}\left(x,z+\frac{h}{2},t\right)\right) \tag{29}$$

$$\boldsymbol{\rho^{II} W^{II}{}_P(x,z,t) = 0} \tag{30}$$

$$\rho^I W^{III}{}_P(x,z,t) = H^1{}_{I,II}\left(x,-z+\frac{h}{2},t\right) - H^1{}_{I,I}\left(x,-z+\frac{h}{2},t\right) + H^2{}_{I,I}(x,z+h/2,t) - H^2{}_{I,II}(x,z+h/2,t) - \left(H^1{}_{II,II}\left(x,-z+\frac{h}{2},t\right) - H^1{}_{II,I}\left(x,-z+\frac{h}{2},t\right)\right) - \left(H^2{}_{I,I}\left(x,z+\frac{h}{2},t\right) - H^2{}_{I,II}\left(x,z+\frac{h}{2},t\right)\right) \tag{31}$$

$$\boldsymbol{\rho^I W^{III}{}_P(x,z,t) = 2H^1{}_{I,II}\left(x,-z+\frac{h}{2},t\right) - H^1{}_{I,I}\left(x,-z+\frac{h}{2},t\right) - H^1{}_{II,II}\left(x,-z+\frac{h}{2},t\right)} \tag{32}$$

Let us develop the integral terms:

$$H^1{}_{i,j}(x,y,\nu,t) = \rho^j\rho^j \int_{\alpha=-\infty}^{\infty}\int_{\beta=-\infty}^{\infty}\int_{\gamma=0}^{\zeta(x,t)} \frac{C_{i,j}}{((\nu-\gamma)^2+(x-\alpha)^2+(y-\beta)^2)^3} dXdYd\gamma \tag{33}$$

We substitute: $X = \alpha - x$ and $dX = d\alpha$. Similarly: $Y = y - \beta$ and $dY = d\beta$; $Z = \gamma - z$ and $dZ = d\gamma$:

$$H^1{}_{i,j}(x,y,\nu,t) = \rho^i\rho^j \int_{Y=-\infty}^{\infty}\int_{X=-\infty}^{\infty}\int_{Z=+\nu}^{\zeta(x,t)+\nu} \frac{C_{i,j}}{((Z)^2+(X)^2+(Y)^2)^3} dXdYdZ \tag{34}$$

Let us integrate along Y first, given that $\zeta(x,t)$ is not dependent on Y :

$$H^1{}_{i,j}(x,\nu,t) = \rho^i\rho^j \int_{X=-\infty}^{\infty}\int_{Z=+\nu}^{\zeta(x,t)+\nu}\int_{Y=-\infty}^{\infty} \frac{C_{i,j}}{((Z)^2+(X)^2+(Y)^2)^3} dXdYdZ \tag{35}$$

For that, we consider $C = \sqrt{X^2+Z^2}$ and do a variable change considering $Y = C.tan(u)$ and $dY = \frac{C}{cos^2(u)}du$:

$$H^1{}_{i,j}(x,\nu,t) = \rho^i\rho^j C_{i,j} \int_{X=-\infty}^{\infty}\int_{Z=+\nu}^{\zeta(x,t)+\nu}\int_{u=-\pi/2}^{\pi/2} \frac{1}{(C)^6(1+tan^2(u))^3}\frac{C}{cos^2(u)} dXdudZ \tag{36}$$

$$\boldsymbol{H^1{}_{i,j}(x,\nu,t) = \frac{3}{8\pi}A_{i,j} \int_{\alpha=-\infty}^{\infty}\int_{\beta=0}^{\zeta(x,t)} \frac{1}{((\nu-\beta)^2+(x-\alpha)^2)^{5/2}} d\alpha d\beta} \tag{37}$$

To obtain the body force, we can derivate along relevant directions:

- Derivative along x

$$-\frac{\partial H^1{}_{i,j}}{\partial x} = \frac{3}{8\pi}A_{i,j}\left[-\frac{\partial\zeta}{\partial x}\int_{\alpha=-\infty}^{\infty}\frac{1}{((\nu-\zeta(x,t))^2+(x-\alpha)^2)^{5/2}}d\alpha d\beta + 5\int_{\alpha=-\infty}^{\infty}\int_{\beta=0}^{\zeta(x,t)}\frac{(x-\alpha)}{((\nu-\beta)^2+(x-\alpha)^2)^{7/2}} d\alpha d\beta\right] \tag{38}$$

- Derivative along z

$$-\frac{\partial H^1{}_{i,j}}{\partial z} = \frac{15}{8\pi}A_{i,j}\left[\int_{\alpha=-\infty}^{\infty}\int_{\beta=0}^{\zeta(x,t)}\frac{\partial\nu}{\partial z}\frac{(\nu-\beta)}{((\nu-\beta)^2+(x-\alpha)^2)^{7/2}} d\alpha d\beta\right] \tag{39}$$

Considering that $\nu = -z + \frac{h}{2}$ for film III:

$$\rho^J W^{III}{}_P(x,z,t) = 2H^1{}_{I,II}\left(x,-z+\frac{h}{2},t\right) - H^1{}_{I,I}\left(x,-z+\frac{h}{2},t\right) - H^1{}_{II,II}\left(x,-z+\frac{h}{2},t\right) \tag{40}$$

- Along the z-axis:

$$-\frac{\partial \rho^{III} W^{III}{}_P(x,z,t)}{\partial z} = -\frac{3}{8\pi}\left(A_{I,I} + A_{II,II} - 2A_{I,II}\right) \int_{\alpha=-\infty}^{\infty} \left[\frac{1}{\left(\left(-z+\frac{h}{2}-\zeta(\alpha,t)\right)^2 + (x-\alpha)^2\right)^{5/2}} - \frac{1}{\left(\left(-z+\frac{h}{2}\right)^2 + (x-\alpha)^2\right)^{5/2}} \right] \quad (41)$$

- Along the x-axis:

$$-\frac{\partial \rho^{III} W^{III}{}_P(x,z,t)}{\partial x} = \frac{3}{8\pi}\left(A_{I,I} + A_{II,II} - 2A_{I,II}\right) \left[\frac{\partial \zeta}{\partial x} \int_{\alpha=-\infty}^{\infty} \frac{1}{\left(\left(-z+\frac{h}{2}-\zeta(\alpha,t)\right)^2 + (x-\alpha)^2\right)^{5/2}} d\alpha d\beta - 5 \int_{\alpha=-\infty}^{\infty} \int_{\beta=0}^{\zeta(x,t)} \frac{(x-\alpha)}{\left(\left(-z+\frac{h}{2}-\beta\right)^2 + (x-\alpha)^2\right)^{7/2}} d\alpha d\beta \right] \quad (42)$$

Considering that $\nu = z + \frac{h}{2}$ for I:

$$\rho^I W^I{}_P(x,z,t) = H^2{}_{I,I}\left(x, z+\frac{h}{2}, t\right) + H^2{}_{II,II}\left(x, z+\frac{h}{2}, t\right) - 2H^2{}_{I,II}\left(x, z+\frac{h}{2}, t\right) \quad (43)$$

- Along the z-axis:

$$-\frac{\partial \rho^I W^I{}_P(x,z,t)}{\partial z} = -\frac{3}{8\pi}\left(A_{I,I} + A_{II,II} - 2A_{I,II}\right) \left[\int_{\alpha=-\infty}^{\infty} \left(\frac{1}{\left(\left(z+\frac{h}{2}-\eta(x,t)\right)^2 + (x-\alpha)^2\right)^{5/2}} - \frac{1}{\left(\left(z+\frac{h}{2}\right)^2 + (x-\alpha)^2\right)^{5/2}} \right) d\alpha \right] \quad (44)$$

- Along x-axis:

$$-\frac{\partial \rho^I W^I{}_P(x,z,t)}{\partial x} = -\frac{3}{8\pi}\left(A_{I,I} + A_{II,II} - 2A_{I,II}\right) \left[\frac{\partial \eta}{\partial x} \int_{\alpha=-\infty}^{\infty} \frac{1}{\left(\left(z+\frac{h}{2}-\eta(x,t)\right)^2 + (x-\alpha)^2\right)^{5/2}} d\alpha - 5 \int_{\alpha=-\infty}^{\infty} \int_{\beta=0}^{\eta(x,t)} \frac{(x-\alpha)}{\left(\left(z+\frac{h}{2}-\beta\right)^2 + (x-\alpha)^2\right)^{7/2}} d\alpha d\beta \right] \quad (45)$$

Total contributions

For any point in film I (top layer):

$$\rho^I W^I{}_{total}(x,z,t) = \rho^I W^I{}_{NP}(x,z,t) + \rho^I W^I{}_P(x,z,t) \quad (46)$$

For any point in Film II:

$$\rho^{II} W^{II}{}_{total}(x,z,t) = \rho^{II} W^{II}{}_{NP}(x,z,t) + \rho^{II} W^{II}{}_P(x,z,t) \quad (47)$$

For any point in Film III (bottom layer):

$$\rho^I W^{III}{}_{total}(x,z,t) = \rho^I W^{III}{}_{NP}(x,z,t) + \rho^I W^{III}{}_{P}(x,z,t) \quad (48)$$

CFD model: introduction

Let us now present the multiphase fluid dynamics (CFD) simulation employing this body force in (non-linear) solving of Navier-Stokes equations. Multi-scale physics at play during fiber thermal drawing are simulated using an interactive script, downscaling a cross-section at each step. The velocity field along the fiber axis is in first approximation assimilated to a plug flow[10]. We discretize in time the simulation, which works in two main steps, as described in the schematic in Supplementary Fig. S21: (i) Direct solving of Navier-Stokes equations determines the evolution of the two film interfaces at constant cross-sectional dimensions between times t and t+dt; (ii) The cross-sectional profile at t+dt is scaled down to account for the drawing process. The simulation then loops between steps (i) and (ii) until the final cross-sectional dimensions are reached or forces diverge. Re-meshing at every time step ensures solver accuracy spanning over a large set of length scales.

CFD model: disjoining pressure models

We run simulations for each disjoining pressure method, for different thicknesses (e.g. 0.4 and 0.5 μm). The effective surface tension option stems from the linearized model. We monitor its Height Ratio R^L_{height} and compare it to that of the general non-linearized body force approach R^{NL}_{height}. The absolute difference is plotted in Supplementary Fig. S6. Although we are fairly in the linearization approximation (varicose amplitude $a_0 \approx 500\, nm \ll \lambda_0, with\, \lambda_0 = 10, 20\, or\, 40\, \mu m$), significant deviation between both approaches appears in the late stages of the draw when the film thickness becomes small. This shows that the classical disjoining pressure expression fails to capture well the dynamics once destabilizing interactions become preponderant.

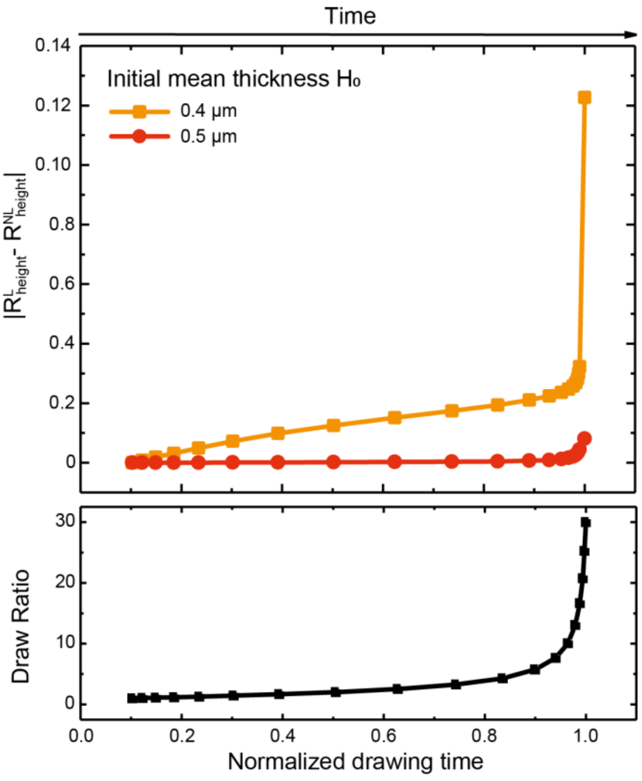

Supplementary Fig. S6 | Height ratio discrepancy evolution for H_0 = 0.4 or 0.5 µm and DR_f = 30 using two distinct methods to incorporate the disjoining pressure: (i) through an effective surface tension and (ii) through a body force approach. Evolution of DR is shown below against normalized drawing time $\frac{t-t_f}{t_0-t_f}$ to show when downscaling is highest, and film thickness lowest.

This difference between disjoining pressure and body force approach can also be found in the critical thickness yielding filamentation. Our simulation results show that as the fiber is scaled down, both linear and general potentials predict an onset to filamentation (e.g. inversion of the Height Ratio curve, not shown here) in the final stage of the process, for sufficiently reduced initial thicknesses. However, while the linearized disjoining pressure shows a filamentation threshold between 400 nm to 500 nm, the non-linear potential shows a transition from presence to absence of filamentation between 500 nm and 600 nm. This highlights the difference between the two disjoining pressure approaches.

Moreover, two important features should be pointed out. First, the system dynamics are highly sensitive to interfacial energy (see Supplementary Note 8). If the actual interaction coefficient was lower than the one calculated, reflow would be highly disfavored and break-up enhanced. Secondly, additional shearing effects occur perpendicularly to the cross-sectional profile studied here[11], which are neglected in our case. Nonetheless, our Lagrangian framework monitoring the evolution of in-plane instabilities during the draw still unravels the rationale behind the process presented in this work, driven by the interplay between disjoining and Laplace pressures.

Note 8. CFD model sensitivity analysis

Mesh and time discretization

To validate the accuracy of the numerical model, we first considered the influence of spatial and time discretization on film dynamics (Supplementary Fig. S7). To assess mesh accuracy, we monitored the film's Height Ratio using the simulation framework for increasing meshing density (decreasing numbers indicate increasing solver accuracy). The limited deviation apparent in the graph highlights that a meshing density of 5 is sufficient for accurate calculations. The system seems significantly more dependent on time discretization. Simulation time is also sensitive to the time discretization step. We set for the rest of this work a time discretization in 20 steps as a compromise between simulation time and convergence of results.

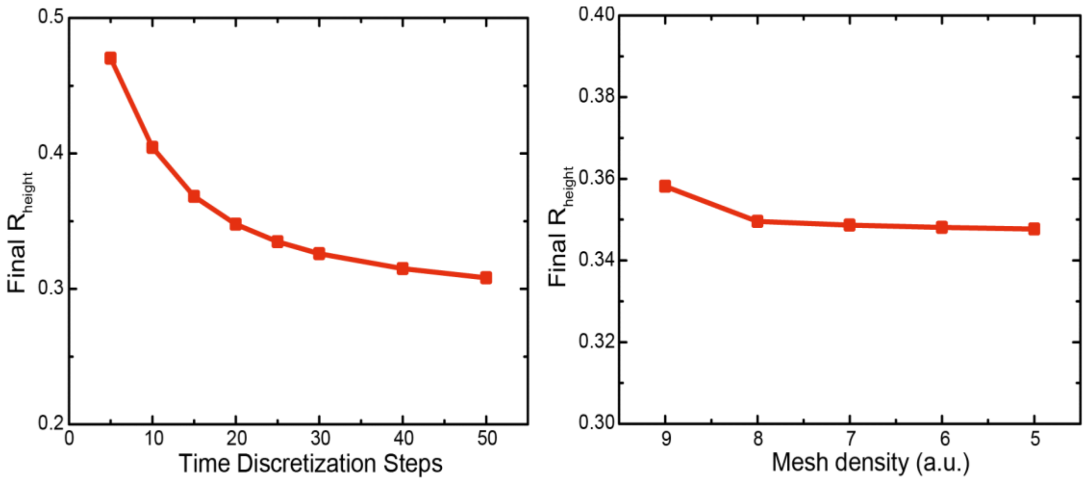

Supplementary Fig. S7 | Final R_{height} obtained for a film of initial thickness H_0 = 1 µm and initial height ratio $R_{height,0}$ = 0.5 as a function of (left) the number of time discretization steps (COMSOL-MATLAB scaling-remeshing steps) and (right) COMSOL mesh density. As a compromise between accuracy and computational requirements, we choose a mesh density of 5 and a time step of 20.

Draw ratio

As in the main text, a study of height ratio dynamics for an identical film thickness and varying final draw ratio DR_f reveals a limited influence of the draw ratio on reflow (Supplementary Fig. S8). Indeed, similar reflow dynamics reflect the similarity of the initial systems, whose final reflow factor essentially depends on the final length scale involved.

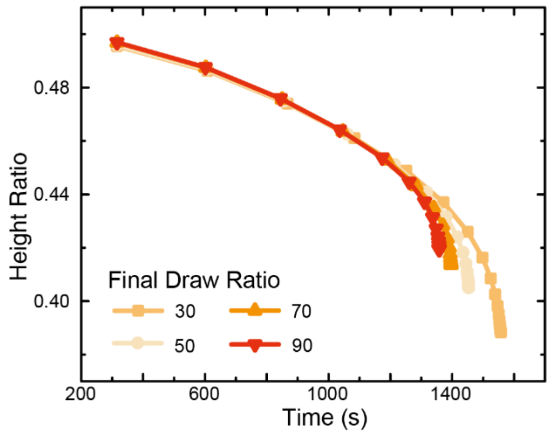

Supplementary Fig. S8 | Influence of increasing draw ratio DR on the height ratio R_{height} evolution as a function of time.

Interfacial energy

Interfacial tension between the polymer matrix and the substrate is a fundamental parameter which largely determines the texture reflow dynamics. In Supplementary Fig. S9, the height ratio is plotted for interfacial tensions increasing from γ_{12}= 3.85 mN/m to γ_{12}= 385 mN/m with DR = 50 and H_0 = 1.5 µm. The reflow is significantly increased for higher surface tension, which leads to completely flat films for interfacial tension of the order of 385 mN/m. The overall system sensitivity to this parameter is an important element of this study, and hence calls for particularly accurate values. In the present case, the interfacial tension coefficient γ_{12} can be estimated based on geometric means[11]:

$$\gamma_{12} = \gamma_1 + \gamma_2 - 2\left[(\gamma_1^d \gamma_2^d)^{1/2} + \left(\gamma_1^p \gamma_2^p\right)^{1/2}\right]$$

with γ_i the surface tension associated to component i = 1 (As_2Se_3) or 2 (PEI), γ_i^d the dispersive component and γ_i^p the polar component of the surface tension associated to component i. In this equation, the interfacial tension of a polymer with similar values for γ_i^d and γ_i^p would be much smaller than the surface tension of each component. In our case, considering the similarity of As and Se atomic electronegativity, we consider that there is no polar component in As_2Se_3. Electronegativity of As and Se atoms are χ_{As}=2.18 and χ_{Se}=2.55 on the Pauling electronegativity scale. Typically, a maximum difference of 0.2 - 0.5 indicates nonpolar covalent, which makes the covalent bonding As-Se a non-polar covalent bond, hence justifying our assumption above. We thus consider

$\gamma_1^p \sim 0$ and $\gamma_1^d \sim \gamma_1$. Based on (ref.[12]), we have $\gamma_1 = 0.1\ N.m^{-1}$; $\gamma_2 = 41.0\ mN.m^{-1}$; $\gamma_2^d = 26.32\ mN.m^{-1}$. The interfacial tension γ_{12} thus amounts to γ_{12} = 38.5 mN/m. This value is significantly lower than the free surface tension of the ChG γ_{11}=100 mN/m, which would be the relevant surface tension parameter in the case of open-air reflow (e.g. exposed film on a substrate). Therefore, the use of a polymer cladding plays a key role in limiting reflow and reducing the varicose mode decay. This is in line with previous observations in purely polymeric systems[13].

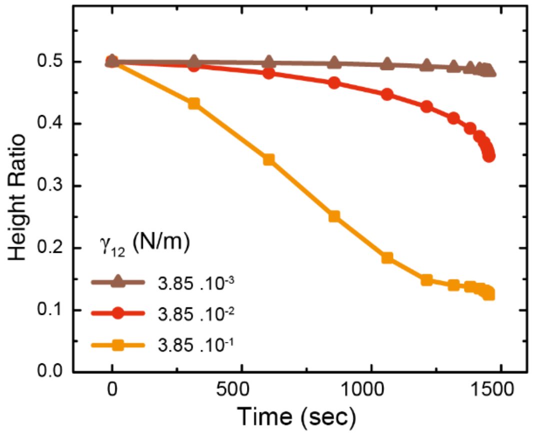

Supplementary Fig. S9 | Evolution of height ratio versus time for increasing interfacial tension.

Film thickness

We further proceeded to compare the evolution in time of R_{height} depending on the initial film thickness H_0. Results of the simulation for this parameter swept from 0.4 µm to 0.8 µm are shown in Fig. S10.

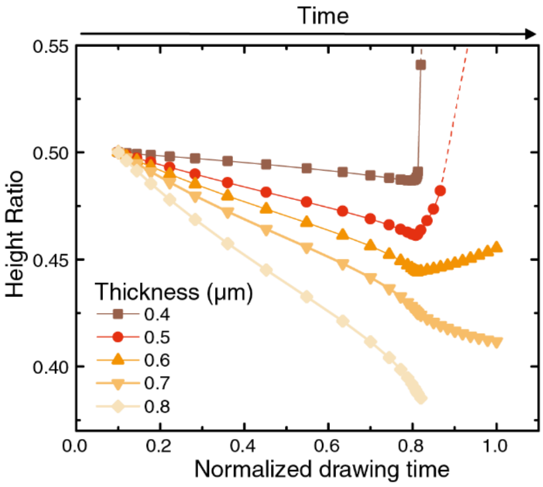

Fig. S10 | Evolution of R_{height} in time for $R_{height,0}$ = 0.5, DR = 30, and different initial mean film thicknesses H_0.

Note 9. Experimental validation of the critical $R_{height,0}$ for filamentation

The initial Height Ratio $R_{height,0} = \frac{H_{max}-H_{min}}{H_{max}+H_{min}}$ for the film can be measured from SEM image analysis, as shown on Supplementary Fig. S11.

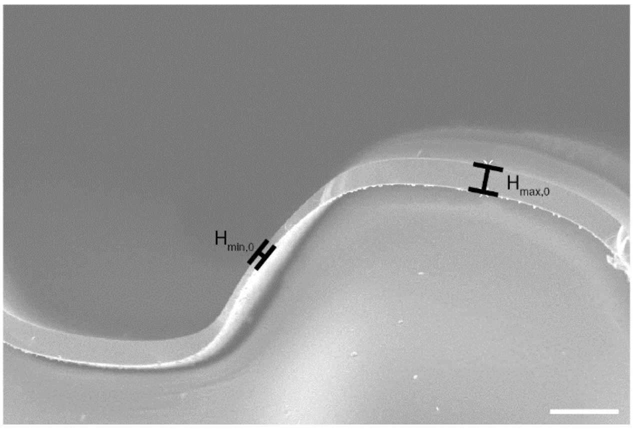

Supplementary Fig. S11 | $H_{min,0}$ and $H_{max,0}$ measured on an SEM image cross section of the initial film before drawing to calculate $R_{height,0}$. Scale bar: 4 µm

To validate experimentally for which value of $R_{height,0}$ filamentation occurs, a glancing angle *gradient* was used for PVD. As shown in Supplementary Fig. S12a, the nanoimprinted templated film was placed on a curved 3D-printed scaffold. Its slope was designed to make an angle θ linearly increasing with x, the distance along the film's width (longitudinal), with respect to the vertical axis (Supplementary Fig. S12b). Clips were also 3D-printed to bend and maintain the film against the curved surface. The whole structure was then placed inside the evaporator chamber for ChG PVD. The film was then placed in a preform and drawn into a fiber, as described in the main text. Microtomy was used to produce SEM image cross-sections of (i) the fiber and (ii) the initial film in different locations (Supplementary Fig. S12c). For H_0 = 550 nm, the initial $R_{height,0}$ was measured from image analysis in nine locations on the film (Supplementary Fig. S12d). A linear regression gives $R_{height,0}$ as function of x. The exact location $\frac{x_{lim}}{DR}$ at which filamentation starts in the fiber (middle SEM images) was measured and correlated to the corresponding critical $R_{height,0}(x_{lim})$ ~ 0.7.

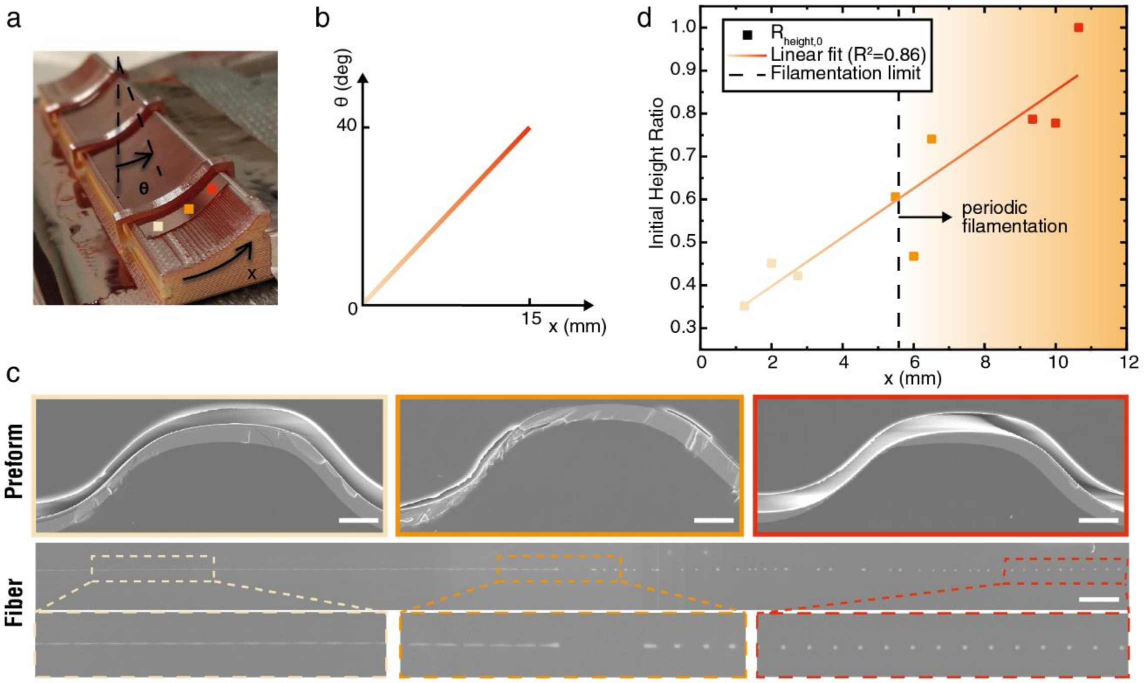

Supplementary Fig. S12 | Experimental validation of the critical $R_{height,0}$. a, A ChG film is evaporated onto a templated polymer substrate held with clips against a curved 3D-printed scaffold. **b**, The scaffold is designed for the angle θ with

respect to the vertical axis to increase linearly with x, the film's longitudinal distance. **c**, (top) SEM images of the initial film's geometry in the preform at some of the locations where $R_{height,0}$ was measured, and (bottom) the film after drawing in the fiber. **d**, Linear regression of $R_{height,0}$ as a function of the position x, allowing experimentally to determine that for $R_{height,0}$ > 0.6, NW formation occurs. Scale bars: preform: 2 µm, fiber: 2.4 µm.

It is interesting to note that our filamentation threshold obtained through numerical modelling rested on the hypothesis of a non-retarded VdW potential, which overestimates the strength of destabilizing Van der Waals interactions typically beyond 10 nm[14]. Turning to the characteristic minimal film thicknesses H_{min}, it appears that the onset of filamentation typically occurs when H_{min} is of the order of 10 nm, i.e. at distance where the retardation effects are not yet pronounced. The reduced discrepancy between retarded and non-retarded VdW potential at such reduced distances could help explain why our experimental and theoretical predictions are well in line.

Note 10. Diameter distribution

We demonstrated how depositing at an angle on a templated substrate allows for capillary-break up anisotropy during thermal drawing. Not only does it enable decoupling diameter and spacing by choosing film thickness and substrate period, but compared to the flat film case described by Xu et al.[2], it provides better control over diameter or spacing regularity. From an SEM cross-section of a fiber, we measured the NWs' diameter distribution using image analysis in ImageJ. The distribution is plotted in Supplementary Fig. S13, showing a standard deviation σ = 9 nm for NW mean diameter m = 252 nm. This process relies on a single fiber draw, the diameter/spacing regularity being on par with NW arrays obtained by redrawing[15]. Although our study did not focus on achieving NWs as thin as possible, a cross-section of a NW array achieved for 50 nm diameters is shown in Supplementary Fig. S14.

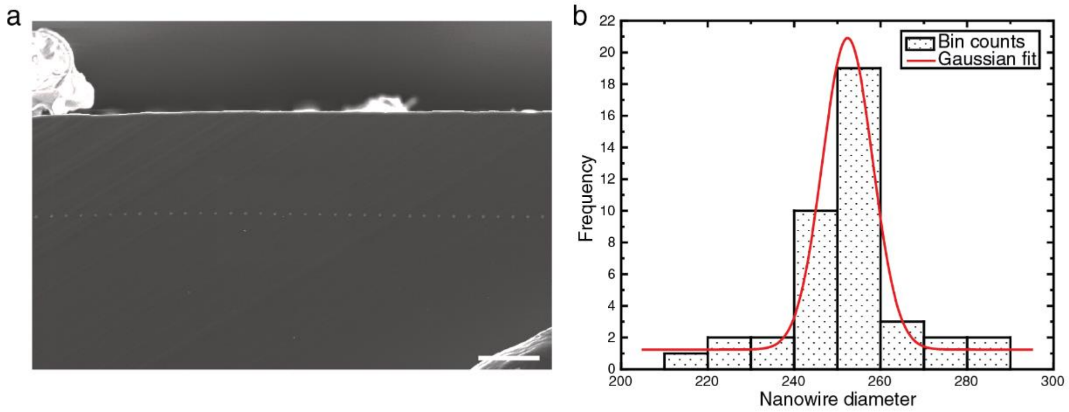

Supplementary Fig. S13 | NW diameter distribution. a, SEM cross section of a NW array inside a fiber used for image analysis. Scale bar: 3.5 µm. **b,** Diameter distribution from image analysis, grouping in 10 nm bins and fitting for a Gaussian distribution (σ = 9 nm, m = 252 nm).

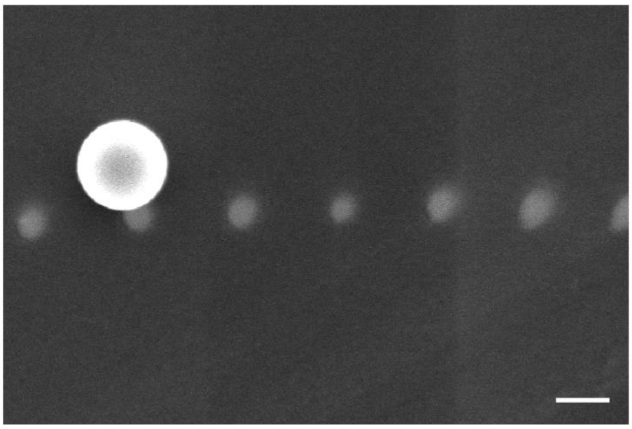

Supplementary Fig. S14 | SEM image cross section of a 50 nm-diameter NW array. Scale bar: 100 nm.

Note 11. Diffraction setup

To measure diffracted beams from the ChG NW array in the fiber, the optical setup depicted on Supplementary Fig. S15 was used. For one measurement, the collimated laser beam is operated at a fixed wavelength λ, with a 1 nm bandwidth. A polarizer is used to control the incident beam's polarization with respect to the fiber. A slit is used to limit the beam to the area where the NWs are, since in the fibers tested, the NW area was approximately 300 µm wide, when the laser beam was around 1 mm in diameter. The slit has an opening of about 100 µm, aligned with the fiber by gluing them together under a microscope in transmission. Finally, transmitted orders are measured at different angles θ using either a spectrometer or a power meter on a rotating stage. The fiber and its slit are mounted together on a multi-axis stage and positioned so that the grating is at the center of the rotating stage. They are also mounted on a goniometer for precise alignment. Indeed, this is required for good horizontality of the diffracted beams, so that the detector's height does not need to be adjusted when measuring at different angles. This is a particularly sensitive requirement for good coupling into a spectrometer device, since it requires focusing into an optical fiber connected to the spectrometer. A lens was used for this purpose, whose focal point was centered on the optical fiber's opening. However, because of some inevitable dispersion in the fiber spacings, some angular dispersion also affects the diffracted beams. This results in some off-optical axis focusing and imperfect coupling into the spectrometer. Thus, we preferred using a power meter without any focusing optics, its 9.5 mm-opening large enough to encompass the whole diffracted order. The distance to the fiber was adjusted to measure only one order at a time.

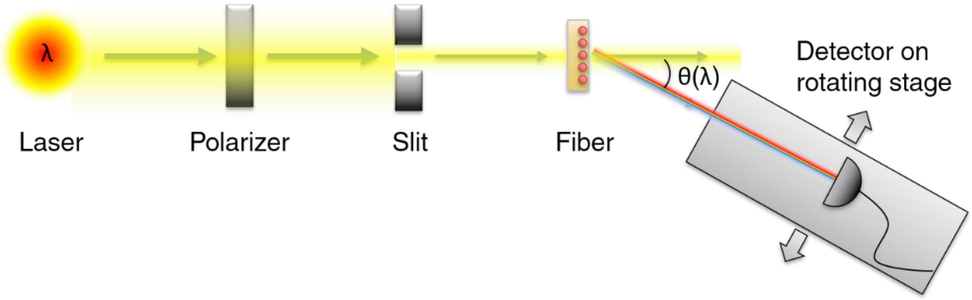

Supplementary Fig. S15 | Diffraction characterization setup, able to measure intensities for beams diffracted at different angles θ(λ)

Note 12. Rigorous coupled-wave analysis (RCWA)

To simulate diffraction behavior for arrays of filaments at various periods, diameters and wavelengths, we used rigorous coupled-wave analysis (RCWA), a semi-analytical method in computational electromagnetics. It is usually employed to predict scattering from periodic dielectric structures. It builds upon a Fourier-space method so that geometries and fields are represented by a sum of spatial harmonics. This method is implemented in a MATLAB environment, based on an open-source package called RETICOLO[16,17]. It is an efficient and accurate toolbox for simulating and describing the electromagnetic field through the grating. We defined a 1D periodic geometry whose unit cell is an As_2Se_3 cylindrical (circular cross sections) filament of diameter d, in a PEI cladding. The geometry must be discretized in slices, as visible in the inset in Supplementary Fig. S16. The top and bottom PEI cladding layers are not considered for simplicity. Refractive indexes for As_2Se_3 were measured with a spectroscopic ellipsometer Sopra GES 5E[1]. Refractive indexes for PEI were taken from literature[18,19]. The simulation calculated absolute efficiencies for the different reflected and transmitted orders for a given geometry, wavelength and number of terms used in the Fourier series expansion of the approximated solution. By looping over wavelengths from 400 to 1000 nm, one can plot efficiencies for all orders, as shown in the example in Supplementary Fig. S16, for instance for d = 328 nm, p = 1.43 µm. For most simulations, we used slices 5 nm thick to reduce computation time. We verified the sensitivity of the results to this parameter by running our script in a simple case for 0.5 nm slices. We estimate this thickness to be much smaller than both wavelength and wire diameter (50 nm at minimum) and thus a reference to compare with. Results for 5 nm slices deviated at most by 1%, when the simulation time was reduced by about a factor 10. We also verified sensitivity to the number of Fourier series terms. This time, the reference taken was 50 terms. Again, we didn’t see more than 1% deviation when using only 15 terms, when the simulation time was reduced by a factor 2.

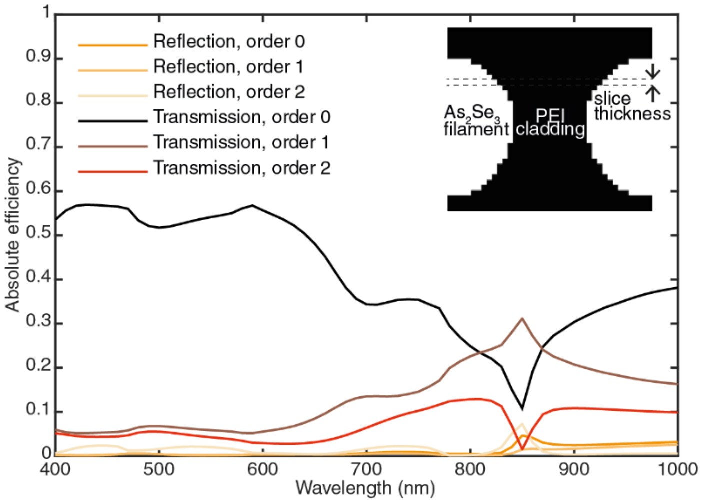

Supplementary Fig. S16 | Simulated absolute efficiencies for the transmitted and reflected orders for d = 328 nm, p = 1.43 µm using RCWA simulations. The inset shows how the unit period geometry was described spatially.

Note 13. Scattering response of NWs

The scattering cross-sections of individual As_2Se_3 NWs of varying diameters, both in air and in PEI, are presented below. We used the Finite Difference Time Domain (FDTD) software Lumerical[20]. The refractive index of As_2Se_3 is taken from our own measurements (see RCWA) and the refractive index of PEI was taken

from literature[18,19]. For a given cylinder radius, scattering cross-sections values were obtained using Lumerical TFSF source (Total-Field Scattered-Field). This broadband plane wave and unit amplitude source is in the form of a 2D square frame surrounding the scattering object - here, an As_2Se_3 disc, representing a cross-section of an infinitely long cylinder. One side of the frame is the injection plane. We chose an injection direction normal to the cylinder axis and a TE-polarization. Regions outside the frame record only the scattered field, by subtracting the incident field contribution to the total field. The scattered power is recorded with monitors surrounding the TFSF source. Normalizing this power with the source intensity yields cross-section values. In PEI, the refractive index contrast is lower than in air, resulting in a broadening of the resonances[21].

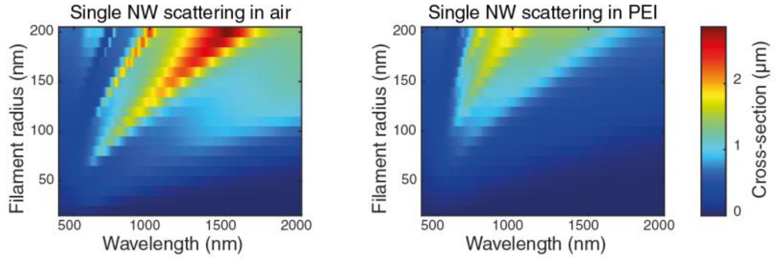

Supplementary Fig. S17 | Scattering cross-section spectra of individual As_2Se_3 NWs of varying diameters, both in air and in PEI, for a polarization parallel to the wires.

We then simulated scattering of *periodically arranged* NWs under normally-incident plane wave illumination. Periodic boundaries were applied in the direction transverse to illumination. Complex transmission coefficients were extracted using an S-parameter analysis[22]. The data shown in Fig. 4 (main text) at λ_i = 800 nm is highlighted by a dashed line in Supplementary Fig. S18. We chose to arrange the NWs with period p = 300 nm, to ensure working in the non-diffraction regime for all wavelengths of interest (500 nm to 1 µm). Both TM and TE polarizations were simulated to verify that although scattering responses differ, phase coverage and high transmission are still present.

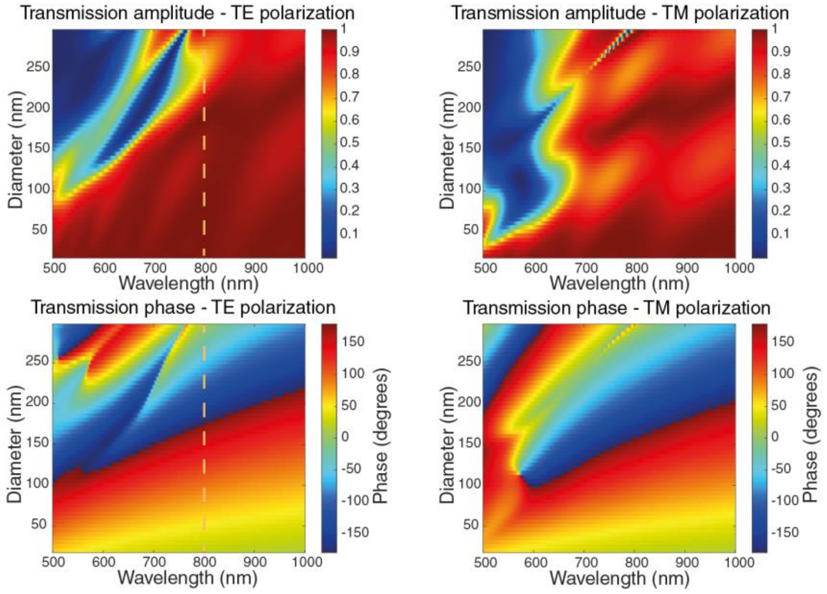

Supplementary Fig. S18 | Scattering response (normalized amplitude and phase) in transmission for a 300 nm-period NW array, for polarizations orthogonal and parallel to the wires.

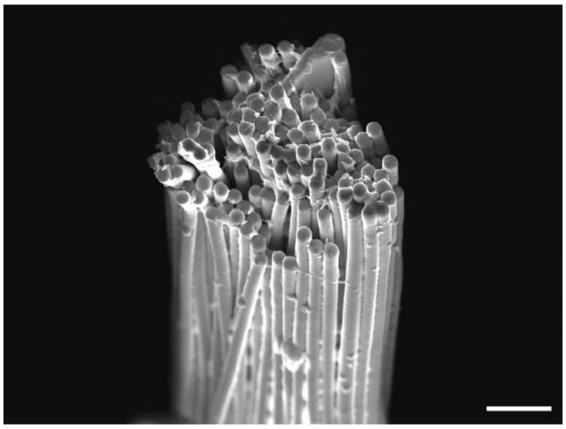

Supplementary Fig. S19 | Filament bundle after dissolving the cladding. Scale bar: 2 µm.

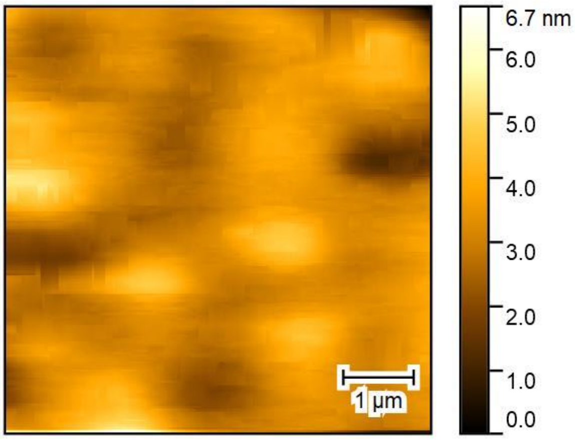

Supplementary Fig. S20 | AFM roughness measurement of an As_2Se_3 film after deposition (and before annealing for preform consolidation) to estimate an upper limit for the initial amplitude of the Rayleigh-type instability.

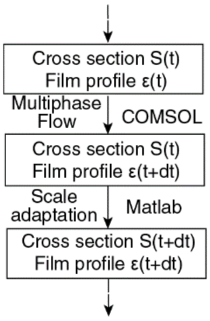

Supplementary Fig. S21 | Schematic illustrating the functioning principle of the time-discretized Lagrangian CFD simulation.

Supplementary references

1. Das Gupta, T. *et al.* Self-assembly of nanostructured glass metasurfaces via templated fluid instabilities. *Nat. Nanotechnol.* **14**, 320–327 (2019).
2. Xu, B. *et al.* Filament formation via the instability of a stretching viscous sheet: Physical mechanism, linear theory, and fiber applications. *Phys. Rev. Fluids* **4**, 073902 (2019).
3. Plateau, J. A. F. *Statique expérimentale et théorique des liquides soumis aux seules forces moléculaires*. (Gauthier-Villars, 1873).
4. Rayleigh, Lord. XVI. On the instability of a cylinder of viscous liquid under capillary force. *The London, Edinburgh, and Dublin Philosophical Magazine and Journal of Science* (1892) doi:10.1080/14786449208620301.
5. Scheid, B., Quiligotti, S., Tran, B. & Stone, H. A. Lateral shaping and stability of a stretching viscous sheet. *Eur. Phys. J. B* **68**, 487–494 (2009).
6. Rayleigh, Lord. On The Instability Of Jets. *Proceedings of the London Mathematical Society* **s1-10**, 4–13 (1878).
7. Jain, R. K. & Maldarelli, C. Stability of Thin Viscoelastic Films with Applications to Biological Membrane Deformation*. *Annals of the New York Academy of Sciences* **404**, 89–102 (1983).
8. Maldarelli, C., Jain, R. K., Ivanov, I. B. & Ruckenstein, E. Stability of symmetric and unsymmetric thin liquid films to short and long wavelength perturbations. *Journal of Colloid and Interface Science* **78**, 118–143 (1980).
9. Horn, R. G. Thin liquid films: Fundamentals and applications. I. B. Ivanov, Ed., Marcel Dekker, Inc., New York, 1988, 1160 pp., $195.00. *AIChE Journal* **35**, 1403–1404 (1989).
10. Page, A. G., Bechert, M., Gallaire, F. & Sorin, F. Unraveling radial dependency effects in fiber thermal drawing. *Appl. Phys. Lett.* **115**, 044102 (2019).
11. FOWKES, F. M. Dispersion Force Contributions to Surface and Interfacial Tensions, Contact Angles, and Heats of Immersion. in *Contact Angle, Wettability, and Adhesion* vol. 43 99–111 (AMERICAN CHEMICAL SOCIETY, 1964).
12. Finch, C. A. Polymer handbook: Third edition Edited by J. Brandrup and E. H. Immergut, Wiley-Interscience, Chichester, 1989. pp. ix + parts I to VIII, price £115·00/$;175·00. ISBN 0–471–81244–7. *British Polymer Journal* **23**, 277–277 (1990).
13. Nguyen-Dang, T. *et al.* Controlled Sub-Micrometer Hierarchical Textures Engineered in Polymeric Fibers and Microchannels via Thermal Drawing. *Advanced Functional Materials* **27**, 1605935 (2017).

14. Israelachvili, J. *Intermolecular and Surface Forces - 3rd Edition*. (Academic Press, 2011).

15. Yaman, M. *et al.* Arrays of indefinitely long uniform nanowires and nanotubes. *Nature Materials* **10**, 494–501 (2011).

16. Hugonin, J. P. & Lalanne, P. RETICOLO software for grating analysis. *arXiv:2101.00901 [physics]* (2021).

17. Hugonin, J.-P. & Lalanne, P. Light-in-complex-nanostructures/RETICOLO: V8. (2020) doi:10.5281/zenodo.3610175.

18. Zhang, X. *et al.* Complex refractive indices measurements of polymers in visible and near-infrared bands. *Appl. Opt., AO* **59**, 2337–2344 (2020).

19. Zhang, X., Qiu, J., Zhao, J., Li, X. & Liu, L. Complex refractive indices measurements of polymers in infrared bands. *Journal of Quantitative Spectroscopy and Radiative Transfer* **252**, 107063 (2020).

20. Nanophotonic FDTD Simulation Software - Lumerical FDTD. *Lumerical* https://www-origin.lumerical.com/products/fdtd/.

21. Groep, J. van de & Polman, A. Designing dielectric resonators on substrates: Combining magnetic and electric resonances. *Opt. Express, OE* **21**, 26285–26302 (2013).

22. Metamaterial S parameter extraction. *Lumerical Support* https://support.lumerical.com/hc/en-us/articles/360042095873-Metamaterial-S-parameter-extraction.
